# Three-dimensional Nanowire Structures for Ultra-Fast Separation of DNA, Protein and RNA Molecules

**DOI:** 10.1038/srep10584

**Published:** 2015-06-15

**Authors:** Sakon Rahong, Takao Yasui, Takeshi Yanagida, Kazuki Nagashima, Masaki Kanai, Gang Meng, Yong He, Fuwei Zhuge, Noritada Kaji, Tomoji Kawai, Yoshinobu Baba

**Affiliations:** 1 Institute of Innovation for Future Society, Nagoya University, JAPAN; 2 FIRST Research Center for Innovative Nanobiodevices, Nagoya University, JAPAN; 3 Department of Applied Chemistry, Graduate School of Engineering, Nagoya University, JAPAN; 4 The Institute of Scientific and Industrial Research, Osaka University, JAPAN; 5 Health Research Institute, National Institute of Advanced Industrial Science and Technology (AIST), JAPAN

## Abstract

Separation and analysis of biomolecules represent crucial processes for biological and biomedical engineering development; however, separation resolution and speed for biomolecules analysis still require improvements. To achieve separation and analysis of biomolecules in a short time, the use of highly-ordered nanostructures fabricated by top-down or bottom-up approaches have been proposed. Here, we reported on the use of three-dimensional (3D) nanowire structures embedded in microchannels fabricated by a bottom-up approach for ultrafast separation of small biomolecules, such as DNA, protein, and RNA molecules. The 3D nanowire structures could analyze a mixture of DNA molecules (50–1000 bp) within 50 s, a mixture of protein molecules (20–340 kDa) within 5 s, and a mixture of RNA molecules (100–1000 bases) within 25 s. And, we could observe the electrophoretic mobility difference of biomolecules as a function of molecular size in the 3D nanowire structures. Since the present methodology allows users to control the pore size of sieving materials by varying the number of cycles for nanowire growth, the 3D nanowire structures have a good potential for use as alternatives for other sieving materials.

Since separation and analysis of biomolecules are essential processes, researchers spend much time carrying out these processes for clarification of biological activities of living things^1^,^2^. Gel filtration chromatography and gel electrophoresis are conventional methods to separate and analyze complex mixtures of biomolecules by charge and size differences of their components^3^. Nowadays, the severity of requirements to separate and analyze DNA, protein, and RNA samples is increasing; however gel electrophoresis has a limitation in its separation range, it requires a large sample volume, and it takes several hours to separate and analyze the sample components^4^,^5^. Capillary electrophoresis using a porous polymer or cellulose in the separation channels has been able to overcome some of the disadvantages, but it still takes several minutes to separate and analyze DNA, protein, and RNA samples and users need highly sophisticated skills and must carry out labor-intensive steps^6^,^7^,^8^,^9^,^10^. Electrophoretic separation by using micro- and nano-structures, called micro- and nano-structure electrophoresis, has attracted interest as a method without any of the above-mentioned disadvantages; the micro- and nano-structures are an alternative to using a sieving material in a microchannel^11^,^12^,^13^,^14^,^15^,^16^,^17^,^18^,^19^,^20^,^21^,^22^,^23^,^24^,^25^,^26^.

To achieve separation and analysis of biomolecules in a short time, Volkmuth and Austin^11^ proposed highly-ordered structures embedded in microchannelsfor separation by size and electrophoretic mobility differences. After them, many research groups decided to use highly ordered structures fabricated by top-down approaches, such as nanopillar arrays^12^,^13^, nanowall arrays^14^, and nanochannels^26^ to simplify and shorten the separation and analysis processes. The nanopillar array of Kaji *et al.*^12^ could separate lambda DNA digested by *Hind III* in 600 s under an applied DC electric field, but it was still difficult to separate DNA molecules smaller than 1 kbp due to the fabricated nanopillar size and spacing limited by electron beam lithography process. Pennathur *et al.*^26^ fabricated a gel-free nanochannel electrophoresis device on glass substrates, and studied the transportation and separation of 10–100 bp dsDNA within 120 s based on the ratio between Debye length and nanochannel depth. Fu *et al.*^15^ could separate denatured protein mixture solutions using chips with continuous flow anisotropic nanofilter arrays. Yasui *et al.*^13^ demonstrated a non-denatured bi-mixture protein separation of trypsin inhibitor (20 kDa) and fibrinogen (340 kDa) based on the EOF mobility difference in a nanopillar array structure device under an applied DC electric field. Since those devices were fabricated by top-down methods, the fabrications themselves were restricted as they required an electron beam lithography step and other sophisticated equipment.

On the other hand, nanostructures fabricated by bottom-up approaches have been suggested to realize separation and analysis of biomolecules in a short time. One example of a bottom-up nanostructures is self-assembled colloidal nanoparticles loaded into microchannels as artificial sieving matrices^22^,^23^,^24^,^25^. Doyle *et al.* 22 loaded the magnetic colloidal nanoparticles into a microfluidic system by the pressure head different of fluid height and controlled forming of the pillar array structures inside microchannels, leading to the separation of λ-DNA and λ-DNA digested by *XhoI* (48.5 kbp, 33.5 kbp and 15 kbp) under an applied DC electric field. Tabuchi *et al.* 23 proposed 30 nm diameter PEG-PLA core shell nanospheres as sieving materials to separate DNA molecules up to 15 kbp within 100 s under an applied DC electric field. Zeng *et al.* 24 obtained good results in separating a multi-mixture of protein molecules (20–205 kDa) by using self-assembled colloidal arrays as a sieving material in microchips. These bottom-up approaches offer many exciting nanostructures as competitors to highly ordered structures fabricated by top-down approaches, however, the bottom-up approaches still have a size limitation for the structures that can be fabricated and low reusability of the fabricated devices compared to the top-down approaches.

Here, we propose to use three-dimensional (3D) nanowire structures fabricated by a bottom-up approach and embedded in microchannels for the ultrafast separation of small biomolecules, such as DNA, protein, and RNA molecules. The proposed embedded nanowire structures have advantages of structures fabricated by both conventional bottom-up and the top-down approaches; in particular, relatively small structures (~10 nm) can be fabricated and the fabricated nanowire structures can be reused many times. Recently, we have reported a self-assembled nanowire array embedded in microchannels for single DNA molecule manipulation and fast separation of relatively large DNA molecules^27^,^28^, however, it is still difficult to separate small biomolecules such as protein and RNA molecules. In the present work, we decreased the pore size in the 3D nanowire structures to a size similar to the hydrodynamic radius of protein and RNA molecules by using a specific number of nanowire growth cycles^28^. This size decrease was something that other approaches could not attain^8^,^12^,^14^,^16^,^21^,^22^,^23^,^24^,^25^,^29^. Since our method offered flexibility and simplicity to control the pore size of sieving material in the microchannels, the separation of DNA, protein, and RNA molecules could be achieved in several tens of seconds.

The 3D nanowire structures were fabricated by employing 7-cycle nanowire growth on fused silica substrates (Fig. 1a). To avoid electrical breakdown under a high applied electric field, we selected fused silica substrates instead of Si substrates. A typical SEM image of the SnO_2_ nanowires (10–20 nm in diameter) is shown in Fig. 1b. In present work, SnO_2_ nanowires backbone, which has a tetragonal rutile structure^30^,^31^ were growth along [100] direction via Au catalyst by vapor liquid solid technique. Then the branches of SnO_2_ nanowire were growth on (001) plane, which has surface energy higher than (010) plane, and the orientation angle is about 90° (Fig.1e and supplementary Fig. S2). When we increased the number of growth cycles to seven, the pore size in the 3D nanowire structures decreased due to branching on the backbone nanowires (Fig. 1c). A 20 nm SiO_2_ layer, as a negatively charge surface on the nanowires, was deposited to avoid the surface charge interaction between biomolecules and nanowire structures. The 3D nanowire structure was measured the pore size in a cross section image and analyzed by FESEM then the histogram of pore size distribution showed that the average pore size was 18.9 ± 8 nm (Fig. 1d). Two important parameters to control for the nanowire devices fabrication are the Au catalyst deposition by DC sputtering which imply to the number of nanowire and the number of nanowire growth cycle which involve to the density of nanowire. We have analyzed the number of nanowire growth for each cycle and we found that the number of nanowire increased approximately 30-50 wires in 1 μm^2^. The volume fraction of nanowire in microchannel was approximately 39.8% that means the pore volume in nanowire area is 60.2% of microchannel. This method allows researchers to control the pore size between nanowires by varying the number of nanowire growth cycles and to select the pore size of the nanowires based on the analytical range of the target biomolecules.

Figure 2a shows an electropherogram for the separation of six small DNA molecules in the 3D nanowire structures, *i.e.* 50, 100, 200, 300, 500 and 1000 bp molecules. The separation resolution and the number of theoretical plates per column length for each DNA molecule are summarized in Table 1. We identified peaks in the electropherograms by comparing the peak position of individual DNA molecule (Fig. 2b). Figure 2c shows the semi-logarithmic plot of DNA mobility against DNA size. There was a linear relationship for small DNA molecules between 50–200 bp. Since the radius of the gyration sizes by Kratky-Porod formular (<*R*_*g*_*>*^*2*^ = *(1/12)b*^*2*^*[(2L/b)-1* *+* *exp(2L/b)]* where *b* is the Khun length of dsDNA and *L* is the DNA contour length) of 50–200 bp DNA molecules are approximately 24.9–59.5 nm, which is a comparable size to the pore size distribution in the 3D nanowire structures (Fig. 1d), the separation regime seems to be the Ogston sieving regime^3^,^32^,^33^. Consequently, the DNA molecules smaller than 200 bp should migrate through the 3D nanowire structures by the Ogston sieving regime, while those larger than 300 bp should migrate through the 3D nanowire structures by a biased reptation mechanism.

Figure 3a shows an electropherogram for the separation of five non-denatured protein molecules in the 3D nanowire structures, *i.e.* trypsin inhibitor (20.1 kDa), protein A (45 kDa), streptavidin (52.8 kDa), β-galactosidase (116 kDa), and fibrinogen (340 kDa). Since the diametric dimensions of the protein molecules are very small (less than 100 nm) and their shapes are very complex, it is a challenge to fabricate structures with a pore size smaller than or similar to the protein molecule size in order to separate the non-denatured protein molecules. The separation resolution and the number of theoretical plates per column length for each protein molecule are summarized in Table 1. We identified the peaks in the electropherograms by comparing the migration time of individual protein molecule and the peak area (Fig. 3b and Supplymentary Figs. S3 and S4). We could observe the different electrophoretic mobilities of each protein molecule in the 3D nanowires structures as a semi-logarithmic plot against protein molecular weight (Fig. 3c). The relationship between the electrophoretic mobility and molecular weight was a log-linear regression (R^2^ = 0.993), which confirmed that the migration behaviors of those proteins were by the Ogston sieving regime^3^,^30^. We also found that the transportation time of each type of protein molecule through the pore (*t*_*transportation*_ = *d/μE,* where *d* is pore size between nanowires, *μ* is electrophoretic mobility and *E* is applied electric field) was shorter than the diffusion time (*t*_*diffusion*_ *=* *d*^*2*^*/D*, where *D* is diffusion coefficient of the respective protein molecule) of each protein molecule (Supplementary Fig. S5b), which also supported migration of protein molecules by the Ogston sieving regime in the 3D nanowire structures^28^.

Figure 4 shows the separation of RNA molecules in the 3D nanowire structures, consisting of 100, 200, 300, 400, 600, 800 and 1,000 bases molecules. It should be emphasized that, to the best of our knowledge, our present method gave the first demonstration of RNA separation in nanostructures within 25 s. The separation resolution and the number of theoretical plates per column length for each RNA molecule are summarized in Table 1. We could carry out the peak identification of RNA molecules by correlating the number of peaks to the gel electrophoresis results (Supplementary Fig. S6).

It should be noted that the 3D nanowire structures could separate DNA, protein, and RNA molecules in a short time. However, the exact separation mechanism of biomolecule in the 3D nanowires still difficult to explain due to the complexity of biomolecule migration behavior and the size of protein or RNA is very small for the individual observation, consequently theoretical and experimental works still require. One scenario to describes our achievement is a relatively large rigidity of the 3D nanowire structures (Young’s modulus ≈ 100 GPa)^34^ and there is a SiO_2_ layer on their surfaces, the applied electric field likely concentrates at the pore spaces between nanowires. Such a highly concentrated electric field would force DNA, protein, and RNA molecules to migrate much faster than in a gel, which has more elasticity (Young’s modulus ≈ 0.1–1 MPa)^35^. The biomolecule migration in a rigid network is like in a free solution environment and that allows a high electric field to be applied for fast separation^27^. On the other hand, the biomolecule migration in a flexible network is retarded by the elasticity of the matrix material so it takes a long time to analyze biomolecules even with the application of a high electric field. Another explanation is the pore size of the 3D nanowire structures small enough to confine DNA, protein, and RNA molecules within them unlike the one or two-dimensional nanostructures fabricated by other top-down approaches. Therefore, we can enhance the number of collisions of DNA, protein, and RNA molecules with the 3D nanowire structures, resulting in good separation resolution and high theoretical plate number.

We have demonstrated that our proposed 3D nanowire structures embedded in microchannels as sieving materials provide fast separation and analysis of biomolecules. Our methodology allows users to control the pore size of the 3D nanowire structures by varying the number of nanowire growth cycles. The 3D nanowire structures showed capabilities to separate complex and very small biomolecules such as DNA, protein and RNA molecules in a short time due to the rigidity of the nanowire structures and their very small pore size which is difficult to attain by other fabrication approaches. The 3D nanowire structures provide an alternative approach to analysis of biomolecules and analytical time saving, consequently our methodology has good potential for commercial mass production and realization of reusable of bio-analytical devices.

## Experiments

### Fabrication of the 3D nanowire structures

Fabrications were carried out using the previously reported procedures^27^,^28^ as Fig. S1. In order to fabricate the 3D nanowire structures, we employed conventional photolithography, electron beam lithography, and vapor-liquid-solid (VLS) growth technique for growth of the nanowires. First of all, a 250 nm Cr layer was deposited on fused silica substrates by RF sputtering (SVC-700LRF, Sanyu Denshi). This layer was prepared as a hard mask for reactive ion etching (RIE). A positive photoresist (TSMR V50, Tokyo Ohka Kogyo Co.) was spin-coated on the substrates. Then, the microchannel pattern with a line width of 25 μm was created by a photolithography process and the Cr layer was etched by Cr etchant solution (H_2_O:Ce(NH_4_)_2_(NO_3_)_6_:HClO_4_, 85:10:5). After that, the microchannel was fabricated by RIE (RIE-10NR, Samco Co.) under CF_4_ gas ambient. The microchannel depth was controlled to be 2 μm. Then, the inlet and outlet via holes (1.5 mm diameter) for the microfluidic system were drilled with an ultrasonic driller (SOM-121, Shinoda Co.). We prepared metal catalysts to define the spatial position of the nanowires within the microchannel. A Cr layer (10 nm thick) was deposited within the microchannel. Positive resist (ZEP520 A7, Zeon Corp.) was coated on the microchannel by spin coating, and then the pattern was drawn by electron beam lithography (SPG-724, Sanyu Electron Co.). After developing the resist, the Cr layer pattern was removed with Cr etchant solution. DC sputtering was used to deposit an Au metal catalyst (particle size 3 nm) within the microchannel for nanowire growth. Then the resist was lifted off by sequential immersion in dimethylformamide, acetone and isopropanol. The SnO_2_ 3D nanowire structures were grown on fused silica microchannel by the gold catalyst-assisted pulse laser deposition (PLD). Once the background pressure of the chamber was evacuated to be 5.0 × 10^−6^ Pa, oxygen and argon mixed gas (1:1000) was introduced into the chamber with the ambient total pressure of 10 Pa. Au film was preheated at the growth temperature 800 °C for 20 minutes then the ArF excimer laser was ablated to SnO_2_ target material for the nanowire growth. After 30 minute, the sample was cool down to room temperature for 30 minutes^36^,^37^,^38^,^39^; these were done more than six times. The Cr layer was lifted off by Cr etchant solution. The SiO_2_ layer was deposited onto the SnO_2_ nanowires using the RF sputtering system to avoid charge interaction between nanowires and biomolecules. Finally, the microchannel was sealed by using a fused silica cover plate (130 μm thick) as described in the literature method^13^,^14^,^27^,^28^.

### DNA, Protein and RNA samples preparation

The mixture of small DNA molecules, which consisted of 50, 100, 200, 300, 500 and 1000 bp molecules, was stained with YOYO-1 (Invitrogen) at a dye base ratio of 1:15 contained in concentrated buffer solution (5 × TBE; 445 mM Tris-Borate and 10 mM EDTA, pH 8.2, Sigma-Aldrich, Inc.). The electrophoretic protein mobility measurements in the nanowire array structure were done by using trypsin inhibitor (Invitrogen), protein A (Invitrogen), streptavidin (Invitrogen), β-galactosidase (Novusbio), and fibrinogen (Invitrogen), stained with Alexa Fluor 488 at a dye molecule per mol ratio of 1:4, 1:5, 1:4, 1:3, and 1:8, respectively. We measured the fluorescence intensity of Alexa Fluor 488 dye molecule in each protein samples by microvolume (UV-VIS) spectrophotometer (Nanodrop, ND-1000) as shown in Fig. S3. All protein molecules were mixed together and 3 × TBE buffer with 4% sodium dodecyl sulfate (SDS) was added to the mixture solution. Then the mixture solution was heated with a block incubator (2720 Thermo Cycler, Applied Biosystem) at 85 °C for 5 min and centrifuged for 15 s. After that, 3 × TBE buffer with 0.1% SDS was added to the mixture solution the concentration varied from 10^−4^ to 10^−6^ M. Kim *et al.* 40 reported that the hydrodynamic diameter of proteins increase about 30% after adding SDS to disrupt the tertiary structure and reduce the protein molecules to a linear structure. We also confirmed the protein mixture solution by observing the bands of the protein molecules mixture solution and the protein markers (10–225 kDa, Novagen) in a SDS polyacrylamic gel (15% e-PAGEL, ATTO Corp.) under the applied voltage of 500 V for 60 min as shown in Fig. S6. For the RNA separation, we used the RNA step ladder 0.1–1 kb (Wako) with 2 × loading dye stained by SYBR Gold (Invitrogen) at a dye base ratio of 1:5 and contained in concentrated buffer solution (5 × TBE; 445 mM Tris-Borate and 10 mM EDTA, pH 8.2, Sigma-Aldrich, Inc.).

### Biomolecules Separation and Fluorescence Detection

An inverted fluorescent microscope (Eclipse TE-300, Nikon) equipped with a high voltage sequencer (HVS448-1500, Lab Smith) was used to apply the DC electric fields and a Hg lamp was used to observe fluorescently stained protein molecules. Fluorescence images were captured with an EB-CCD camera (C7190-43, Hamamatsu Photonics K.K.) through a 10 × /1.40 NA objective lens (Nikon). The images were recorded on a DV tape (DSR-11, Sony) and then analyzed by image-processing software (Cosmos 32, Library, Tokyo, Japan).

## Additional Information

**How to cite this article**: Rahong, S. *et al.* Three-dimensional Nanowire Structures for Ultra-Fast Separation of DNA, Protein and RNA Molecules. *Sci. Rep.* **5**, 10584; doi: 10.1038/srep10584 (2015).

## Supplementary Material

Supplementary Information

## Figures and Tables

**Figure 1:**
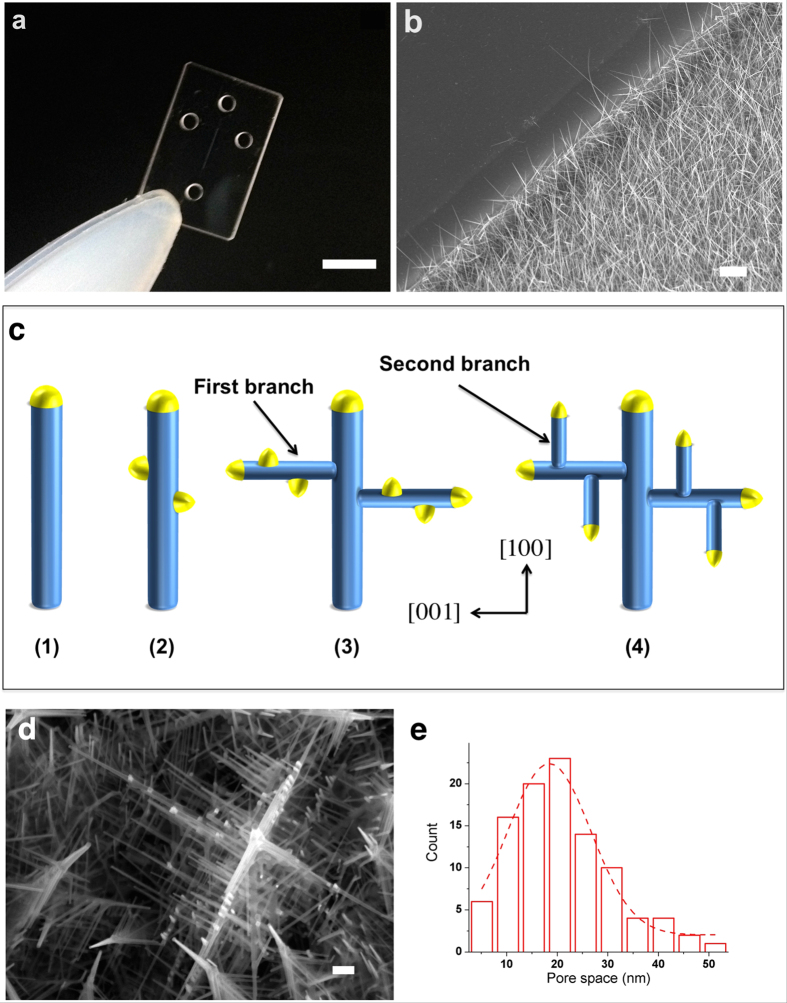
3D nanowire structures. (**a**) Photograph of a device for the 3D nanowire structures; scale bar 5 mm. (**b**) SEM image of SnO_2_ nanowires embedded in a microchannel; scale bar 1 μm. (**c**) Schematic of Au catalyst assisted VLS 3D nanowire growth; nanowire backbone were growth in [100] direction (1), then Au catalyst decorate along nanowire backbone (2), after that, the first nanowire branches growth in [001] direction (3), Au catalysts were deposited on the first nanowire branches and the second nanowire branches were growth by VLS technique as a cycle (4). (**d**) SEM image of the 3D nanowire structures; scale bar 100 nm. (e) Pore size distribution in the 3D nanowire structures.

**Figure 2:**
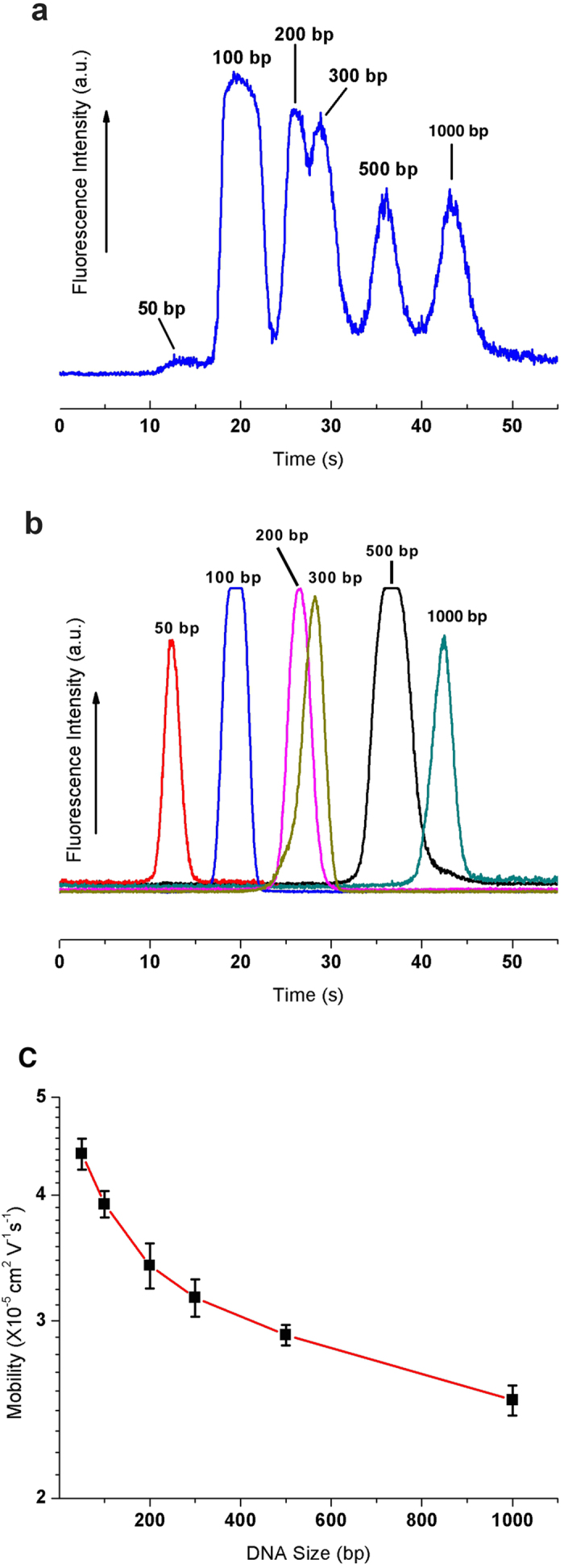
Separation and mobility of DNA molecules in the 3D nanowire structures. (**a**) Separation of 50 bp (40 ng/μL), 100 bp (30 ng/μL), 200 bp (30 ng/μL), 300 bp (30 ng/μL), 500 bp (30 ng/μL) and 1000 bp (30 ng/μL) molecules in the 3D nanowire structures. The electropherograms were obtained at 500 μm from the entrance of the 3D nanowire structures. The applied electric field in the separation channel was 100 V/cm. (**b**) The electropherogram of each type of DNA molecule to verify the migration time of each separation peak (*E* = 100 V/cm, *L* = 500 μm). (**c**) Semi-log plot of electrophoretic mobility as a function of DNA size under the applied electric field of 100V/cm.

**Figure 3:**
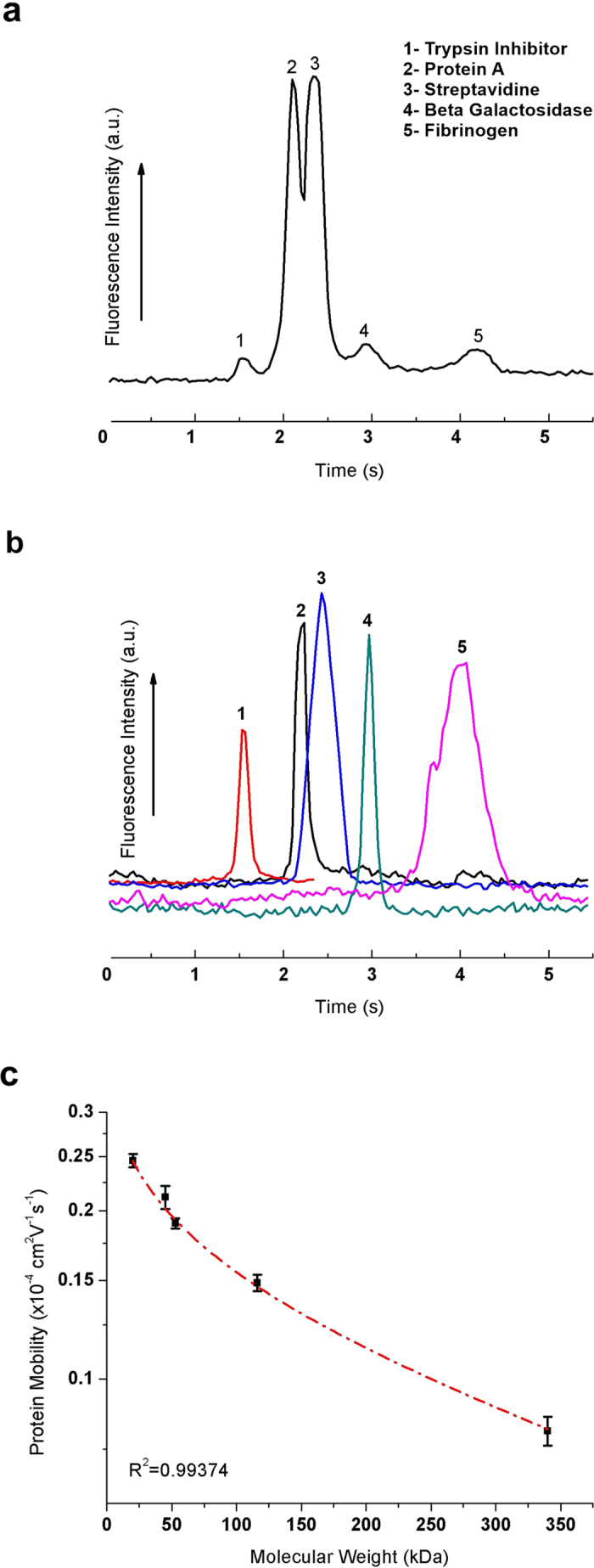
Separation and mobility of protein molecules in the 3D nanowire structures. (**a**) Separation of **(1)** trypsin inhibitor (20.1 kDa), **(2)** protein A (45 kDa), **(3)** streptavidin (52.8 kDa), **(4)** β-galactosidase (116 kDa) and **(5)** fibrinogen (340 kDa). The electropherograms were obtained at 2000 μm from the entrance of the 3D nanowire structures. The applied electric field in the separation channel was 500 V/cm. (**b**) The electropherograms of each type of protein molecule to verify the migration time of each separation peak (*E* = 2000 V/cm, *L* = 500 μm). (**c**) Semi-log plot of electrophoretic mobility as a function of molecular weight under the applied electric field of 500V/cm.

**Figure 4:**
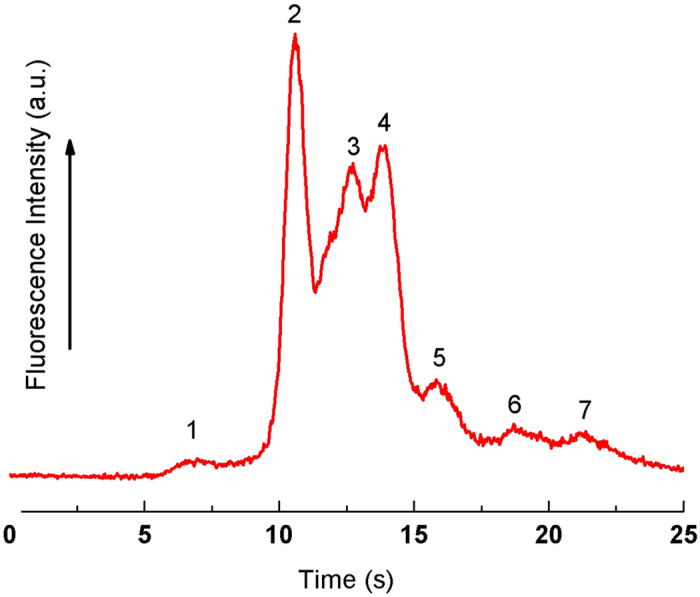
Separation of 0.1–1 kb RNA molecules in the 3D nanowire structures. The electropherogram was obtained at 250 μm from the entrance of the 3D nanowire structures. The applied electric field in the separation channel was 300 V/cm.

**Table 1:**
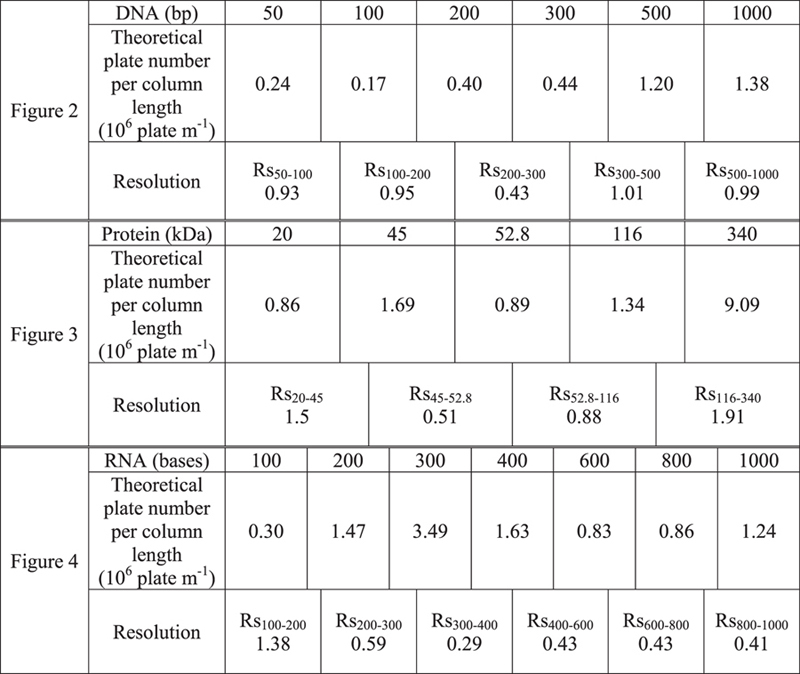
Separation resolution and theoretical plate number per column length in Figures 2, 3 and 4.

## References

[b1] Watson J. & Crick F. Molecular structure of nucleic acids. Nature 171, 738–739 (1953). 13054692 10.1038/171737a0

[b2] Maxam A. M. & Gilbert W. A new method for sequencing DNA. Proc. Natl. Acad. Sci. USA 24, 99–103 (1992). 1422074

[b3] Viovy J. Electrophoresis of DNA and other polyelectrolytes: Physical mechanisms. Rev. Mod. Phys. 72, 813–872 (2000).

[b4] Heller C., Duke T. & Viovy J. Electrophoretic mobility of DNA in gels. II. Systematic experimental study in agarose gels. Biopolymers 34, 249–259 (1994).

[b5] Semenov A., Duke T. & Viovy J. Gel electrophoresis of DNA in moderate fields: The effect of fluctuations. Phys. Rev. E. 51, 1520–1537 (1995). 10.1103/physreve.51.1520 9962796

[b6] Chang H. & Yeung E. S. separation of DNA by capillary electrophoresis. J. Chromatogr. B 669, 113–123 (1995). 10.1016/0378-4347(95)00044-j 7581875

[b7] Gao Q. & Yeung E. S. A Matrix for DNA Separation: Genotyping and Sequencing Using Poly(vinylpyrrolidone) Solution in Uncoated Capillaries. Anal. Chem. 70, 1382–1388 (1998). 9553496 10.1021/ac970999h

[b8] Huang M.-F., Kuo Y.-C., Huang C.-C. & Chang H.-T. Separation of long double-stranded DNA by nanoparticle-filled capillary electrophoresis. Anal. Chem. 76, 192–196 (2004). 14697050 10.1021/ac034908u

[b9] Li Z., Dou X., Ni Y., Sumitomo K. & Yamaguchi Y. The influence of polymer concentration, applied voltage, modulation depth and pulse frequency on DNA separation by pulsed field CE. J. Sep. Sci. 33, 2811–2817 (2010). 20715140 10.1002/jssc.201000188

[b10] Li Z., Dou X., Ni Y. & Yamaguchi Y. Separation of long DNA fragments by inversion field capillary electrophoresis. Anal. Bioanal. Chem. 401, 1661–1667 (2011). 21766216 10.1007/s00216-011-5228-4

[b11] Volkmuth W. & Austin R. DNA electrophoresis in microlithographic arrays. Nature 358, 600–602 (1992). 1501715 10.1038/358600a0

[b12] Kaji N. *et al.* Separation of long DNA molecules by quartz nanopillar chips under a direct current electric field. Anal. Chem. 76, 15–22 (2004). 14697027 10.1021/ac030303m

[b13] Yasui T. *et al.* Electroosmotic flow in microchannels with nanostructures. ACS Nano 5, 7775–7780 (2011). 21902222 10.1021/nn2030379

[b14] Yasui T. *et al.* DNA separation in nanowall array chips. Anal. Chem. 83, 6635–6640 (2011). 21770422 10.1021/ac201184t

[b15] Fu J., Schoch R. B., Stevens A. L., Tannenbaum S. R. & Han J. A patterned anisotropic nanofluidic sieving structure for continuous-flow separation of DNA and proteins. Nat. Nanotechnol. 2, 121–128 (2007). 18654231 10.1038/nnano.2006.206 PMC2621439

[b16] Han J. & Craighead H. G. Characterization and optimization of an entropic trap for DNA separation. Anal. Chem. 74, 394–401 (2002). 11811414 10.1021/ac0107002

[b17] Salieb-Beugelaar G. B., Dorfman K. D., van den Berg A. & Eijkel J. C. T. Electrophoretic separation of DNA in gels and nanostructures. Lab chip. 9, 2508–2523 (2009). 19680576 10.1039/b905448k

[b18] Dorfman K. D. DNA electrophoresis in microfabricated devices. Rev. Mod. Phys. 82, 2903–2947 (2010).

[b19] Kaji N., Okamoto Y., Tokeshi M. & Baba Y. Nanopillar, nanoball, and nanofibers for highly efficient analysis of biomolecules. Chem. Soc. Rev. 39, 948–956 (2010). 20179817 10.1039/b900410f

[b20] Dorfman K., King S. & Olson D. Beyond gel electrophoresis: Microfluidic separations, fluorescence burst analysis, and DNA stretching. Chem. Rev. 113, 2584–2667 (2013). 23140825 10.1021/cr3002142 PMC3595390

[b21] Park S.-G., Olson D. W. & Dorfman K. D. DNA electrophoresis in a nanofence array. Lab Chip 12, 1463–1470 (2012). 22388662 10.1039/c2lc00016d PMC3508065

[b22] Doyle P. S., Bibette J., Bancaud A. & Viovy J.-L. Self-assembled magnetic matrices for DNA separation chips. Science 295, 2237; 10.1126/science.1068420 (2002). 11910102

[b23] Tabuchi M. *et al.* Nanospheres for DNA separation chips. Nat Biotechnol. 22, 337–340 (2004). 14990956 10.1038/nbt939

[b24] Zeng Y. & Harrison D. J. Self-assembled colloidal arrays as three-dimensional nanofluidic sieves for separation of biomolecules on microchips. Anal. Chem. 79, 2289–2295 (2007). 17302388 10.1021/ac061931h

[b25] Minc N., Fu C., Dorfman K. D. & Gosse C. Quantitative Microfluidic Separation of DNA in Self-Assembled Magnetic Matrixes. Anal. Chem. 76, 3770–3776 (2004). 15228353 10.1021/ac035246b

[b26] Pennathur S. *et al.* Free-solution oligonucleotide separation in nanoscale channels. Anal. Chem. 79, 8316–8322 (2007). 17883279 10.1021/ac0710580

[b27] Yasui T. *et al.* DNA Manipulation and Separation in Sublithographic-Scale Nanowire Array. ACS Nano 7, 3029–3035 (2013). 23484881 10.1021/nn4002424

[b28] Rahong S. *et al.* Ultrafast and wide range analysis of DNA molecules using rigid network structure of solid nanowires. Sci. Rep. 4, 5252; 10.1038/srep05252 (2014). 24918865 PMC5381479

[b29] Li Z. *et al.* Capillary electrophoresis of a wide range of DNA fragments in a mixed solution of hydroxyethyl cellulose. Anal. Methods 6, 2473–2477 (2014).

[b30] Leonardy A., Hung W. Z., Tsai D. S., Chou C. C. & Huang Y. S. Structural features of SnO2 nanowires and raman spectroscopy analysis. Cryst. Growth Des. 9, 3958–3963 (2009).

[b31] Baur W. H. Über die Verfeinerung der Kristallstrukturbestimmung einiger Vertreter des Rutiltyps: TiO 2 , SnO 2 , GeO 2 und MgF 2. Acta Crystallogr. 9, 515–520 (1956).

[b32] Ogston A.G., The space in a uniform random suspension of fibres. Trans. Faraday Soc. 54, 1754–1757 (1958).

[b33] Benoit H. & Doty P. Light scattering from non gaussian chains. J. Phys. Chem. 57, 958–963 (1953).

[b34] Barth S., Harnagea C., Mathur S. & Rosei F. The elastic moduli of oriented tin oxide nanowires. Nanotechnology 20, 115705; 10.1088/0957-4484/20/11/115705 (2009). 19420453

[b35] Kolahi K. S. *et al.* Effect of substrate stiffness on early mouse embryo development. PLoS One 7, e41717; 10.1371/journal.pone.0041717 (2012). 22860009 PMC3409240

[b36] Klamchuen A. *et al.* Study on transport pathway in oxide nanowire growth by using spacing-controlled regular array. Appl. Phys. Lett. 99, 193105; 10.1063/1.3660246 (2011).

[b37] Meng G. *et al.* Facile and scalable patterning of sublithographic scale uniform nanowires by ultra-thin AAO free-standing membrane. RSC Adv. 2, 10618–10623 (2012).

[b38] Meng G. *et al.* Impact of preferential indium nucleation on electrical conductivity of vapor-liquid-solid grown indium-tin oxide nanowires. J. Am. Chem. Soc. 135, 7033–7038 (2013). 23581597 10.1021/ja401926u

[b39] Meng G. *et al.* A flux induced crystal phase transition in the vapor-liquid-solid growth of indium-tin oxide nanowires. Nanoscale 6, 7033–7038 (2014). 24842296 10.1039/c4nr01016g

[b40] Kim K. H., Lee J. Y. & Moon M. H. Effect of sodium dodecyl sulfate on protein separation by hollow fiber flow field-flow fractionation. Analyst 136, 388–392 (2011). 20963232 10.1039/c0an00172d

