# Supplementary material for: Three-dimensional Nanowire Structures for Ultra-Fast Separation of DNA, Protein and RNA Molecules
Source: Sci Rep. 2015 Jun 15;5:10584. doi: 10.1038/srep10584 (PMC4466590; doi:10.1038/srep10584)
Supplement: Supplementary Information [file srep10584-s1.pdf]

Supplementary information for

Three-dimensional Nanowire Structures for Ultra-Fast Separation of DNA, Protein and RNA Molecules

Sakon Rahong[a,b]*, Takao Yasui[b,c]*, Takeshi Yanagida[d], Kazuki Nagashima[d], Masaki Kanai[d], Gang Meng[d], Yong He[d], Fuwei Zhuge[d], Noritada Kaji[b,c], Tomoji Kawai[d] and Yoshinobu Baba[b,c,e]*

[a]*Institute of Innovation for Future Society, Nagoya University, JAPAN*

[b]*FIRST Research Center for Innovative Nanobiodevices, Nagoya University, JAPAN*

[c]*Department of Applied Chemistry, Graduate School of Engineering, Nagoya University, JAPAN*

[d]*The Institute of Scientific and Industrial Research, Osaka University, JAPAN*

[e]*Health Research Institute, National Institute of Advanced Industrial Science and Technology (AIST), JAPAN*

*Corresponding authors: (S. Rahong) Phone: +81-52-789-3560; Fax: +81-52-789-4666; E-mail: sakon.rahong@apchem.nagoya-u.ac.jp; (T. Yasui) Phone: +81-52-789-4611; Fax: +81-52-789-4666; E-mail: yasui@apchem.nagoya-u.ac.jp; (Y. Baba) Phone: +81-52-789-4664; Fax: +81-52-789-4666; E-mail: babaymtt@apchem.nagoya-u.ac

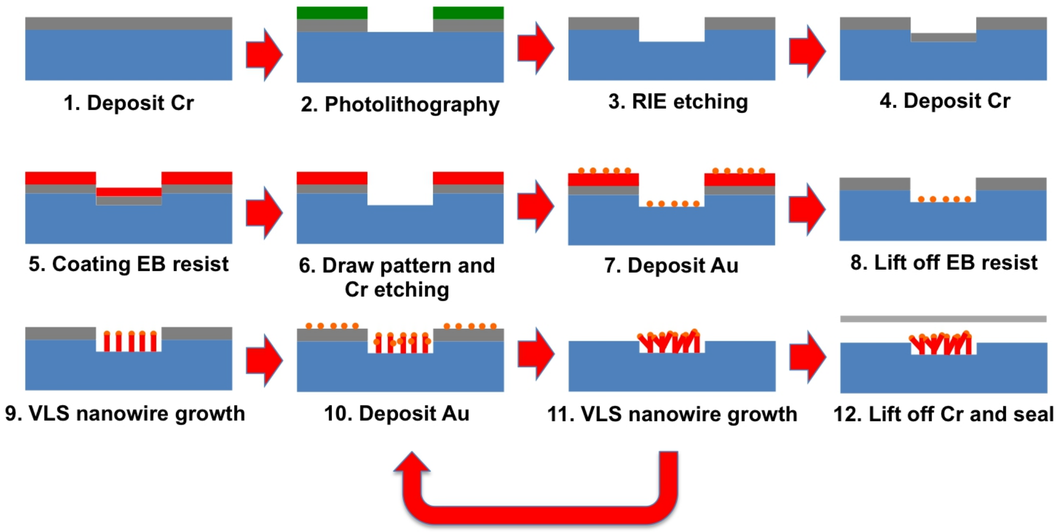

Figure S1. Fabrication process of 3D nanowire structure. (1) A 250 nm Cr layer is prepared on fused quartz substrate by RF sputtering. (2) The microchannel pattern is prepared by a photolithography process and the Cr layer is etched with Cr etchant solution. (3) After that, the substrate is etched 2 μm depth by the RIE process and (4) a 20 nm Cr layer is prepared by RF sputtering. (5) After that, an electron beam resist coating is applied, (6) the nanowire area is drawn by electron beam lithography and the Cr layer is lifted off with the Cr etchant solution to open the area for the nanowire growth. (7) Then, a 3 nm Au layer is deposited as a catalyst for nanowire growth on the substrate by DC sputtering, (8) the electron beam resist is lifted off with dimethylformamide (DMF) solution, leaving the Au catalyst in the microchannel pattern. (9) The nanowires are grown in the microchannel using a pulse laser deposition (PLD) system and (10) a 3 nm Au layer is deposited as a catalyst for nanowire growth on the substrate by DC sputtering and return to growth nanowire as step (9) again as a cycle more 6 times. (11) The Cr layer is lifted off with Cr etchant solution. (12) Then, the 7-cycle growth nanowire device is sealed with a fused quartz cover glass using hexafluorosilisic acid (H_2SiF_6) at 75 °C for 24 h.

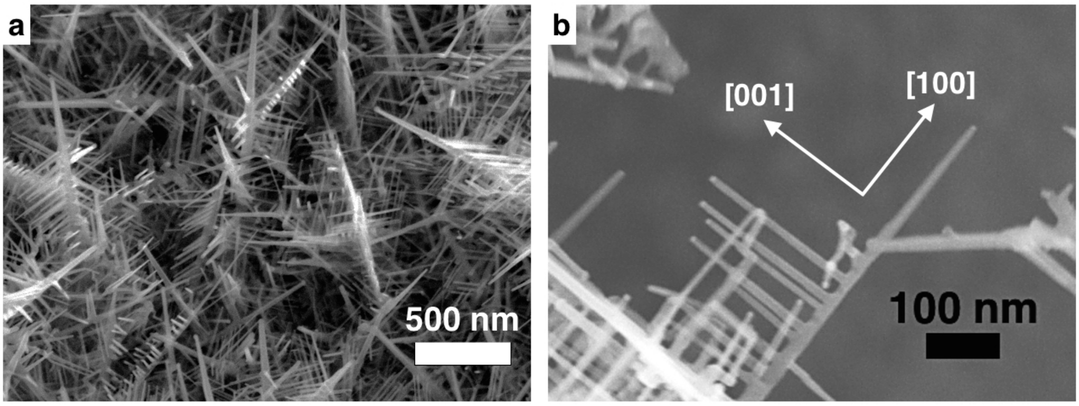

Figure S2. 3D nanowire structure. a) SEM image of 3D nanowire at 7-cycle time nanowire growth and b) The growth direction of SnO_2 nanowire backbone is [100] which is perpendicular with the growth direction of nanowire branches [001].

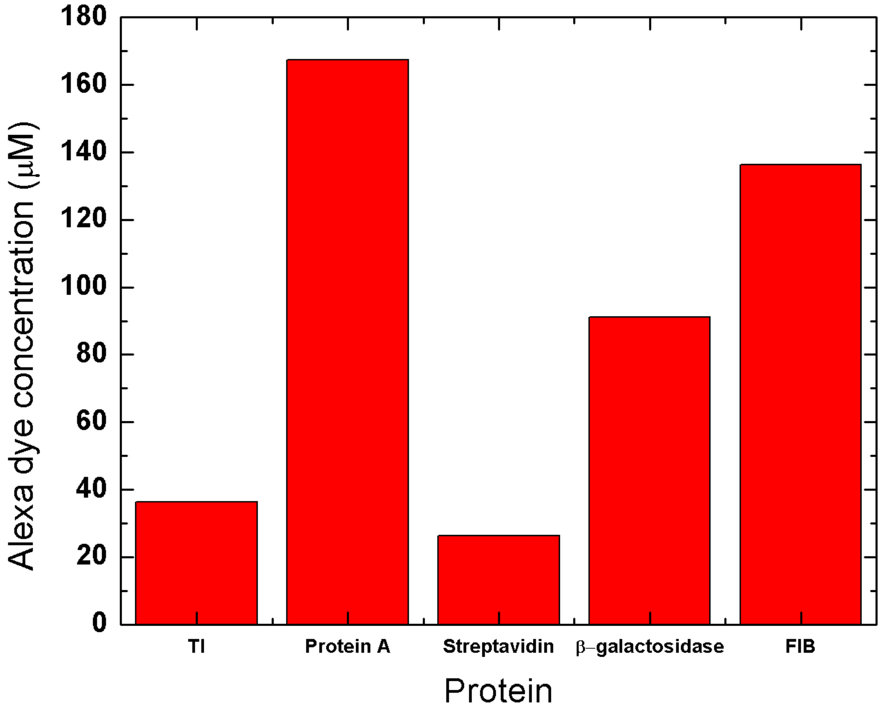

Figure S3. Dye concentration of proteins sample. We measured the Alexa Fluor 488 dye molecule in each protein samples by microvolume (UV-VIS) spectrophotometer (Nanodrop, ND-1000).

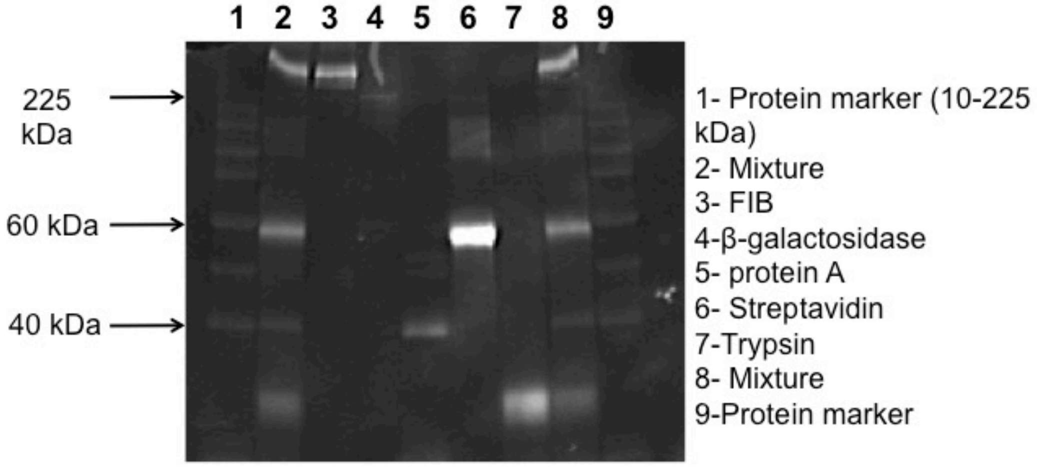

Figure S4. Separation of the protein mixture solution by sodium dodecyl sulfate (SDS) polyacrylamic gel electrophoresis. The protein marker, mixture solution, trypsin inhibitor, streptavidin, protein A, β-galactosidase and fibrinogen were separated in the SDS polyacrylamic gel with an applied electric field of 500 V/cm for 60 min.

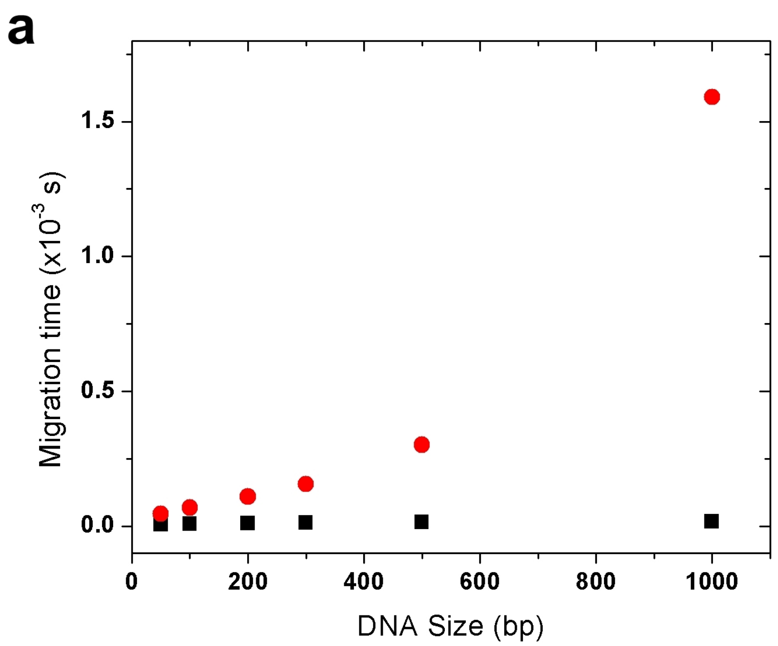

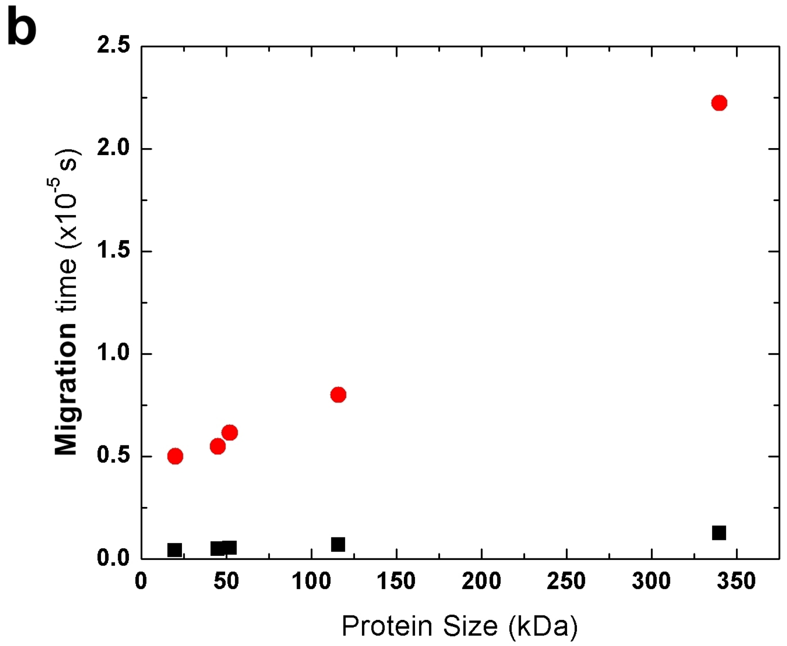

Figure S5. (a) The plot of the diffusion time (red dots, $t_{diffusion} = d^2/D$) and the transportation time (black squares, $t_{transportation} = d/\mu E$) of DNA molecules in the 3D nanowire structures. (b) The plot of the diffusion time (red dots) and the transportation time (black squares) of protein molecules in the 3D nanowire structures.

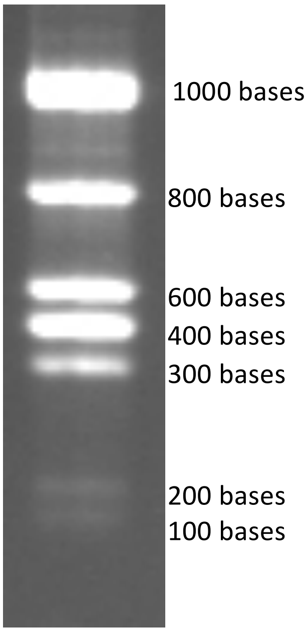

Figure S6. Separation of RNA molecules in gel electrophoresis. The RNA molecules (100-1000 bases) stained by SYBR Gold dye molecule and applied to 2% native agarose gel.
